# Single-crystalline metal-oxide dielectrics for top-gate 2D transistors

**DOI:** 10.1038/s41586-024-07786-2

**Published:** 2024-08-07

**Authors:** Daobing Zeng, Ziyang Zhang, Zhongying Xue, Miao Zhang, Paul K. Chu, Yongfeng Mei, Ziao Tian, Zengfeng Di

**Affiliations:** 1 State Key Laboratory of Materials for Integrated Circuits, Shanghai Institute of Microsystem and Information Technology, Chinese Academy of Sciences, Shanghai, China; 2 Center of Materials Science and Optoelectronics Engineering, University of Chinese Academy of Sciences, Beijing, China; 3 Department of Physics, Department of Materials Science and Engineering, and Department of Biomedical Engineering, City University of Hong Kong, Kowloon, China; 4 Department of Materials Science, Fudan University, Shanghai, China

**Keywords:** Electronic devices, Two-dimensional materials

## Abstract

Two-dimensional (2D) structures composed of atomically thin materials with high carrier mobility have been studied as candidates for future transistors^1–4^. However, owing to the unavailability of suitable high-quality dielectrics, 2D field-effect transistors (FETs) cannot attain the full theoretical potential and advantages despite their superior physical and electrical properties^3,5,6^. Here we demonstrate the fabrication of atomically thin single-crystalline Al_2_O_3_ (c-Al_2_O_3_) as a high-quality top-gate dielectric in 2D FETs. By using intercalative oxidation techniques, a stable, stoichiometric and atomically thin c-Al_2_O_3_ layer with a thickness of 1.25 nm is formed on the single-crystalline Al surface at room temperature. Owing to the favourable crystalline structure and well-defined interfaces, the gate leakage current, interface state density and dielectric strength of c-Al_2_O_3_ meet the International Roadmap for Devices and Systems requirements^3,5,7^. Through a one-step transfer process consisting of the source, drain, dielectric materials and gate, we achieve top-gate MoS_2_ FETs characterized by a steep subthreshold swing of 61 mV dec^−1^, high on/off current ratio of 10^8^ and very small hysteresis of 10 mV. This technique and material demonstrate the possibility of producing high-quality single-crystalline oxides suitable for integration into fully scalable advanced 2D FETs, including negative capacitance transistors and spin transistors.

## Abstract (web summary)

By using intercalative oxidation techniques, stable, stoichiometric and atomically thin single-crystalline Al_2_O_3_ films can be produced, which can be effectively used as a dielectric in top-gated field-effect transistors based on two-dimensional materials.

## Main

As silicon (Si) field-effect transistors (FETs) approach the fundamental limits in scaling, new generations of semiconducting channels are required to reduce the short-channel effects^1,2^. Two-dimensional (2D) materials such as molybdenum disulfide (MoS_2_), which is atomically thin and has high carrier mobility, thus have huge potential in future transistors^4,8–10^. Although 2D materials have better physical and electrical properties than Si, suitable high-quality dielectric materials are not available. Therefore, FET devices based on 2D materials cannot fulfil the full potential predicted theoretically^5,6^. Amorphous oxide dielectrics that work well in Si technology, for example, SiO_2_, Al_2_O_3_ and HfO_2_, cannot provide a uniform and well-defined interface with 2D materials because of the disruption of long-range orders^11–13^. Considering the amorphous nature and ill-defined interfaces, there are difficulties in eliminating charge scattering and traps, thus resulting in (1) high gate leakage current (*J* > 1.5 × 10^−2^ A cm^−2^), (2) high interface state density (*D*_it_ > 10^10^ cm^−2^ eV^−1^) and (3) low dielectric strength (*E*_bd_ < 10 MV cm^−1^), which cannot meet the requirements stipulated by the International Roadmap for Devices and Systems (IRDS)^3,5,7^. Moreover, owing to the inert dangling-bond-free surface of 2D materials, depositing an atomically thin oxide without damaging the adjacent layer remains challenging^6^.

Compared with amorphous oxides, crystalline dielectric materials such as hexagonal boron nitride (hBN)^14^, calcium fluoride (CaF_2_) (ref. ^11^) and perovskite strontium titanium oxide (SrTiO_3_) (ref. ^13^) have atomically flat surfaces that bond well for smoother dielectric/2D material interfaces. In theory, these materials can overcome the problems of interface quality and defect bands. However, there are some disadvantages of crystalline dielectrics. For example, because of the relatively narrow bandgap and low permittivity, hBN with an ultrathin physical thickness exhibits extremely high leakage currents (*J* > 10^3^ A cm^−2^) (ref. ^15^). The use of CaF_2_ and SrTiO_3_ is limited to back-gate FETs^11,13^, although large-scale integrated circuits require top-gate 2D FETs. The unique advantage that Bi_2_SeO_5_ offers is inherently tied to its semiconducting counterpart, which means it may not provide the same benefits when paired with other 2D materials^16^. Other crystalline dielectrics, such as mica, have difficulty in precisely controlling the area and thickness of insulators generated by exfoliation or chemical growth methods^17^. Furthermore, it is challenging to attain an interface state density of 10^10^ cm^−2^ eV^−1^ from devices comprising crystalline dielectrics. Moreover, wafer-scale synthesis of high-quality crystalline dielectrics is quite challenging^11,13,16,17^. As there is still no clear strategy on how to scale dielectrics down to be atomically thin as required by commercial FETs^5,6^, it may be necessary to identify radically different approaches for 2D devices.

Atomically thin metal oxides have attracted attention recently because of their unique electronic, optical and magnetic properties that are rarely found in their bulk counterparts^18,19^. By adopting simple oxidation, a stable, stoichiometric and atomically thin oxide layer can be formed on the metal surface for 2D devices^18,19^. So far, oxides made of transition metals such as HfO_2_, TiO_2_, Fe_2_O_3_ and Ni_2_O_3_; post-transition metals such as Al_2_O_3_; and rare earth metals, including Gd_2_O_3_, have been proposed^18,19^. These atomically thin metal oxides have adequate dielectric properties and atomically flat surfaces, making them suitable for electrostatic modulation of the channels of 2D materials to overcome the present limitations.

In this study, a single-crystalline Al_2_O_3_ (c-Al_2_O_3_) is achieved as a high-quality dielectric layer on the 2D MoS_2_ FET. By combining epitaxial lift-off and intercalative oxidation, an atomically thin c-Al_2_O_3_ with a thickness of 1.25 nm is prepared, which is thinner than the conventional oxides used in advanced Si transistors^20,21^. Owing to the favourable crystalline structure and well-defined interfaces, the gate leakage current (*J* < 1 × 10^−6^ A cm^−2^), interface state density (*D*_it_ = 8.4 × 10^9^ cm^−2^ eV^−1^) and dielectric strength (*E*_bd_ = 17.4 MV cm^−1^) of the atomically thin c-Al_2_O_3_ can meet the IRDS requirements for low-power devices^3,5^. By using the van der Waals (vdW) transfer method, the entire FET stack, including the source, drain, dielectric and gate, can be transferred to the MoS_2_ channel in a one-step process to produce a 2D FET with excellent contact and dielectric interfaces. The top-gate MoS_2_ FET shows a steep subthreshold swing (SS) of 61 mV dec^−1^, ultrahigh on/off current ratio of 10^8^ and small hysteresis of 10 mV. Excellent processing reproducibility and uniformity are demonstrated by fabricating a batch of 100 devices.

**Fig. 1:**
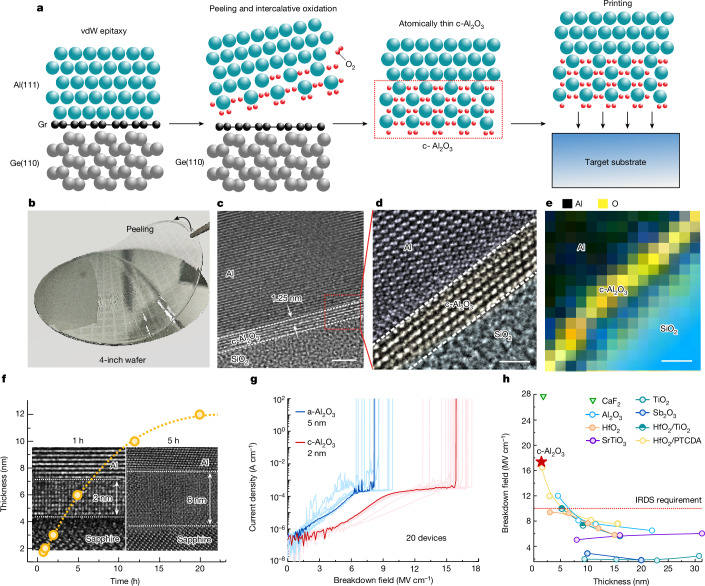
Fabrication and characterization of c-Al_2_O_3_. **a**, Fabrication of atomically thin c-Al_2_O_3_ through epitaxial lift-off and intercalative oxidation of a single-crystalline Al film. **b**, Optical image of the lift-off of Al/c-Al_2_O_3_ from a 4-inch Gr/Ge wafer. **c**, A cross-sectional HR-TEM image of Al/c-Al_2_O_3_ on a target SiO_2_ substrate. **d**, Magnified atomic-resolution image from the red box in **c**. **e**, EEL mapping of Al/c-Al_2_O_3_/SiO_2_. **f**, The relationship between exposure time in an oxygen-deficient environment (0.2 ppm O_2_) and the thickness of c-Al_2_O_3_. Insets, the cross-sectional HR-TEM image of c-Al_2_O_3_ with a thickness of 2 nm and 6 nm obtained by 1 h oxidation and 5 h oxidation, respectively. **g**, The breakdown field of c-Al_2_O_3_ and a-Al_2_O_3_. **h**, The breakdown field compared with film thickness for various dielectrics. Scale bars, 2 nm (**c**); 1 nm (**d**,**e**). Source data

**Extended Data Fig. 1:**
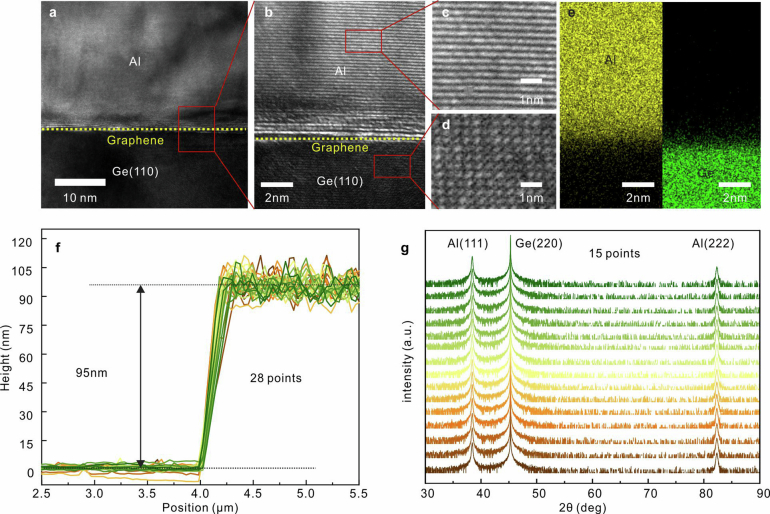
Epitaxial single-crystalline Al (111) on Gr/Ge (110) substrate. a, Cross-sectional HRTEM image of Al/Gr/Ge (110) interface. b, Magnified cross-sectional HRTEM of Al/Gr/Ge (110) interface from the red boxes in (a). c, and d, Magnified cross-sectional HRTEM of single-crystalline Al (111) and single-crystalline Ge (110) remote epitaxial template from the red boxes in (b), respectively. e, EDS mapping images of Al/Gr/Ge interface. f, AFM thickness of single crystalline Al layer grown on Gr/Ge (110) substrate obtained from 28 random points on the wafer. g, High-resolution XRD of Al(111)/Gr/Ge(110) heterostructure obtained from 15 random points on the wafer.

**Extended Data Fig. 2:**
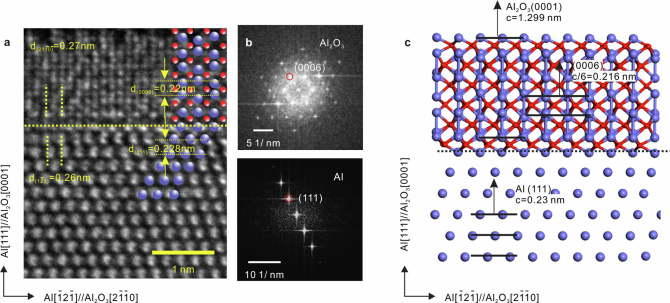
The epitaxial relationship between c-Al_2_O_3_ (0001) and Al (111). a, Cross-sectional HRTEM of Al(111)/Al_2_O_3_(0001). b, Selected area electron diffraction patterns of Al_2_O_3_(0001) and Al(111). c, Side view of Al(111)/ Al_2_O_3_(0001).

**Extended Data Fig. 3:**
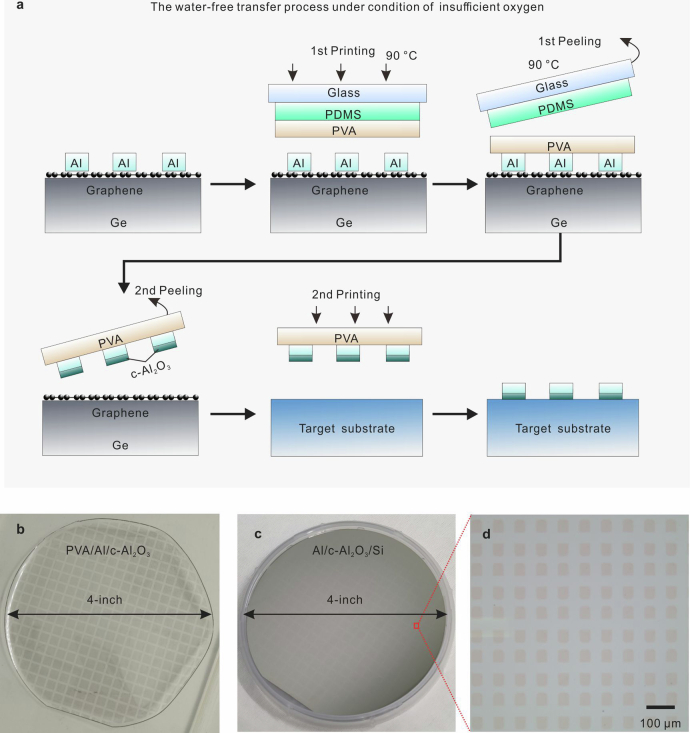
Schematic and optical images of the graphene-assisted Al/c-Al_2_O_3_ water-free transfer printing process. a, Schematic of the water-free transfer process of Al/c-Al_2_O_3_ patterns under an oxygen-deficient environment in a glovebox. The process starts with a clean CVD Gr/Ge substrate; followed by defining Al patterns by electron beam lithography (EBL)/photolithography, depositing Al by electron beam evaporation and lifting off; first printing dry PVA film on Al patterns using a PDMS stamp at 90 °C; second peeling off Al patterns from Gr/Ge substrate using dry PVA film at room temperature in a glovebox; second transferring and printing PVA/Al/c-Al_2_O_3_ on target substrate using a PDMS stamp at 90 °C; finally, the PVA film is dissolved by dimethyl sulfoxide (DMSO). b, Optical image of Al/c-Al_2_O_3_ patterns transferred onto a 4-inch PVA film. c, Optical image of Al/c-Al_2_O_3_ patterns transferred onto a 4-inch Si wafer. d, Magnified optical image of Al/c-Al_2_O_3_ patterns from the red box in (c).

**Extended Data Fig. 4:**
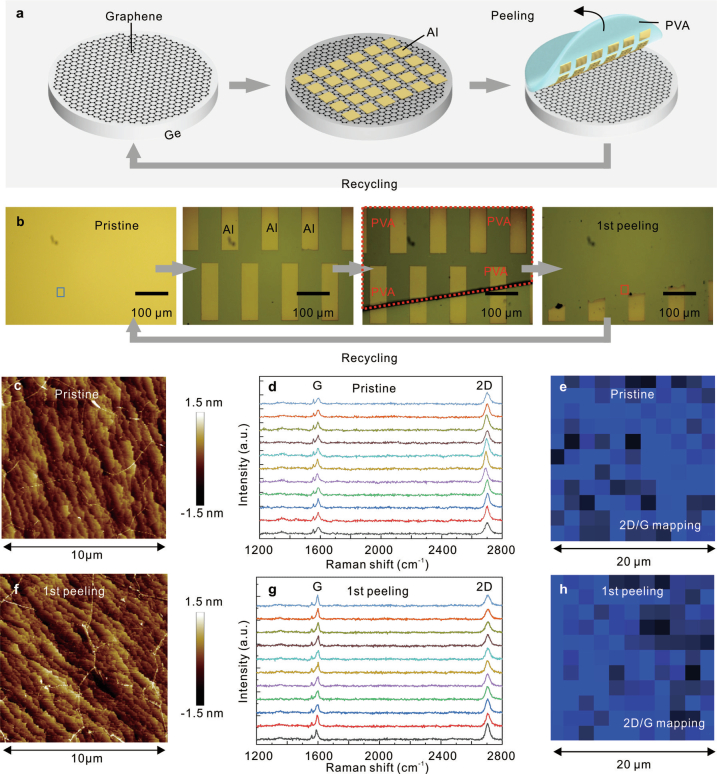
The reuse of Gr/Ge template substrate. a, and b, Schematic and optical images of reusing Gr/Ge template substrate for Al/c-Al_2_O_3_ patterns transfer. c, AFM image of pristine Gr/Ge substrate with a surface roughness of 0.261 nm from the blue box in (b). d, and e, Raman line mapping and area mapping of pristine Gr/Ge substrate. f, AFM image of Gr/Ge substrate from the red box in (b) after first peeling with the surface roughness of 0.299 nm. g, and h, Raman line mapping and area mapping of Gr/Ge substrate from the red box in (b) after first peeling.

Figure 1a shows a scalable method to synthesize high-quality atomically thin c-Al_2_O_3_ layers without complex chemistry or sophisticated equipment. A graphene (Gr)/germanium (Ge) wafer is used as the template, and wafer-scale single-crystalline Al is produced by electron beam evaporation using the vdW epitaxy approach^22–24^. Given that Gr/Ge substrates can be manufactured up to 12 inches in diameter considering the epitaxy of Ge on Si, the industrial-scale production of single-crystalline Al becomes a tangible possibility. The cross-sectional high-resolution transmission electron microscopy (HR-TEM) image and X-ray diffraction results are shown in Extended Data Fig. 1 to confirm the wafer-scale vdW epitaxy of single-crystalline Al(111) on single-crystalline Gr/Ge(110). We proved that a patterned metal with an atomically flat surface can be delaminated from graphene by exploiting the weak vdW force^25^. In this case, Al can be peeled off readily from graphene and then oxidized mildly at room temperature in the 0.2-ppm oxygen environment. Owing to the atomically flat single-crystalline Al surface and oxygen-deficient environment (0.2 ppm O_2_), homogeneous coverage of chemisorbed oxygen atoms and limited penetration of oxygen can be attained. Consequently, a depolarized c-Al_2_O_3_ layer is formed when one oxygen atom chemisorbs on an Al atom, which has been confirmed theoretically and experimentally^19^. In only a few seconds, an ultrathin c-Al_2_O_3_ layer with a thickness of several nanometres is formed by the layer-by-layer mechanism. The cross-sectional HR-TEM image presented in Extended Data Fig. 2 shows the epitaxial relationship between c-Al_2_O_3_ (0001) and Al (111). Finally, the atomically thin c-Al_2_O_3_ is transferred onto a target substrate such as SiO_2_, MoS_2_ or Au to fabricate the device. The fabrication details are presented in the Methods and Extended Data Fig. 3. To prevent Al from oxidizing in H_2_O, the entire process is water-free. Moreover, benefiting from the water-free transfer process, the graphene surface is not damaged thereby allowing repeated use of the Gr/Ge wafer (Extended Data Fig. 4).

Figure 1b shows that the lift-off Al/c-Al_2_O_3_ has close to 100% yield as evidenced by delamination of the 4-inch patterned Al film from the Gr/Ge substrate using polyvinyl alcohol; Supplementary Video 1 shows the entire peeling process. The lifted-off c-Al_2_O_3_ films are transferred onto the SiO_2_ substrate, and the cross-sectional HR-TEM image is shown in Fig. 1c. The thickness of the c-Al_2_O_3_ layer on the Al surface is about 1.25 nm. The magnified atomic-resolution image of the red box in Fig. 1c is shown in Fig. 1d disclosing the sandwiched structure of Al/c-Al_2_O_3_/SiO_2_. Electron energy loss spectroscopy (EEL) mapping shows the elemental distributions (Fig. 1e), and the distributions of Al and O are in accordance with the sandwiched structure.

**Extended Data Fig. 5:**
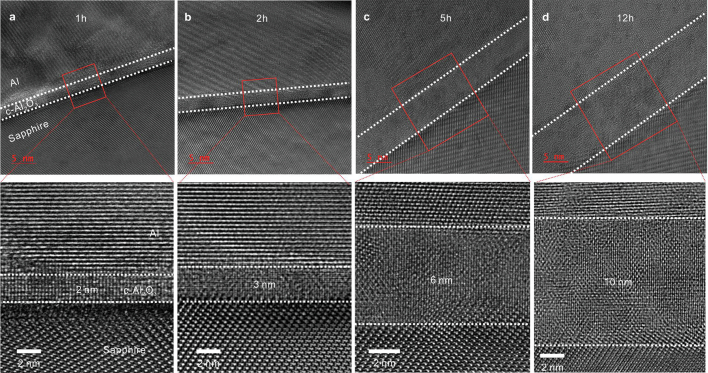
Cross-sectional HRTEM images of vdW-transferred Al/c-Al_2_O_3_ with different thickness on the sapphire substrate. a-d, The cross-sectional HRTEM images and corresponding enlarged images from the red box of 2 nm, 3 nm, 6 nm, and 10 nm c-Al_2_O_3_ by exposing Al to an oxygen-deficient environment for 1 h, 2 h, 5 h, and 12 h, respectively.

Moreover, the thickness of c-Al_2_O_3_ can be manipulated by exposing the vdW epitaxial Al layer to an oxygen-deficient environment for various durations. As shown in Fig. 1f, as the oxidation time is increased from 1 h to 12 h, the oxidation rate decreases from 2 nm h^−1^ to 0.8 nm h^−1^. This is because the oxidation process is controlled by the interface reaction at first and then by oxygen diffusion^16^. Figure 1f (insets) shows the cross-sectional HR-TEM image of c-Al_2_O_3_ with thicknesses of 2 nm (oxidation for 1 h) and 6 nm (oxidation for 5 h), and more HR-TEM images are shown in Extended Data Fig. 5. The results confirm that it is possible to prepare c-Al_2_O_3_ layers with different thicknesses by controlling the oxidation time. Moreover, the thickness mapping of the 4-inch c-Al_2_O_3_/Al wafer is shown in Supplementary Fig. 1 and Supplementary Table 1, which indicates the exceptionally uniform coefficient of variation of ±6%.

Figure 1g shows the breakdown field (*E*_bd_) compared with the current density trend. c-Al_2_O_3_ remains completely insulating for the operating fields well above the IRDS requirement of 10 MV cm^−1^. The average *E*_bd_ determined from 20 metal–insulator–metal (MIM) devices with the c-Al_2_O_3_ insulator is 16 MV cm^−1^ and the maximum is 17.4 MV cm^−1^. In comparison, the amorphous Al_2_O_3_ (a-Al_2_O_3_) layer with a thickness of 5 nm fabricated by lifting off the Al film in an ambient environment exhibits a small average *E*_bd_ of about 8 MV cm^−1^ similar to the previous studies^26,27^. The enhancement of *E*_bd_ may arise from the energy gap increment or the defect density reduction in crystalline insulators^5,28^. The *E*_bd_ versus film thickness for different metal oxides is compared in Fig. 1h, and the details are provided in Supplementary Table 2. Owing to the crystalline structure and large bandgap (8.8 eV) (refs. ^26,28^), defect-assisted tunnelling in c-Al_2_O_3_ is suppressed. As a result, the atomically thin c-Al_2_O_3_ exhibits one of the highest *E*_bd_ (17.4 MV cm^−1^) compared with amorphous oxides (TiO_2_ (ref. ^29^), Al_2_O_3_ (ref. ^30^) and HfO_2_ (refs. ^31–33^) as well as single-crystalline oxides (CaF_2_ (ref. ^34^), SrTiO_3_ (ref. ^13^) and Sb_2_O_3_ (ref. ^12^)). It is important to note that *E*_bd_ of c-Al_2_O_3_ exceeds the IRDS requirement for low-power devices (*E*_bd_ > 10 MV cm^−1^) (refs. ^3,5^).

**Fig. 2:**
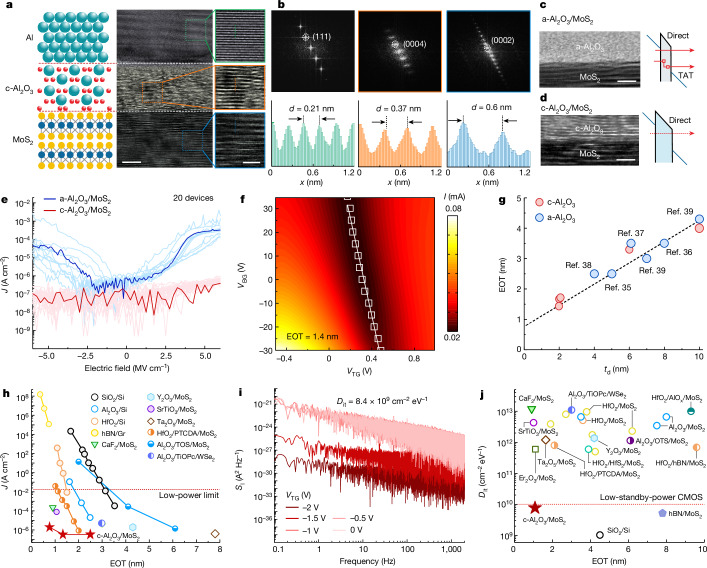
Properties of Al/c-Al_2_O_3_ gate. **a**, Schematic (left), cross-sectional HR-TEM (middle) and magnified atomic-resolution (right) images of an Al/c-Al_2_O_3_/MoS_2_ heterostructure. **b**, Selected area electron diffraction patterns (top) obtained from the dashed green, orange and blue boxes in **a**. Line intensity profiles (bottom) along the dashed green, orange and blue lines in **a**. **c**, HR-TEM image of a-Al_2_O_3_/MoS_2_ (left) and the important tunnelling contributions (right). **d**, HR-TEM image of c-Al_2_O_3_/MoS_2_ (left) and the important tunnelling contributions (right). **e**, Experimental gate leakage currents for 2D FETs with c-Al_2_O_3_ and a-Al_2_O_3_. **f**, The relationship between current and gate voltage. *V*_TG_, top-gate voltage; *V*_BG_, back-gate voltage. **g**, The relationship between EOT and dielectric thickness. **h**, Experimental gate leakage currents compared with EOT measured at standard operating gate voltages of 1 V. **i**, Noise power spectra as a function of frequency. **j**, Comparison of *D*_it_ values measured for Si devices and different 2D technologies. Scale bars, 5 nm (**a**, bottom row, middle); 2 nm (**a**, bottom row, right); 2 nm (**c**,**d**). TAT, trap-assisted tunnelling. Source data

The gate leakage currents (*J*) and interface state densities (*D*_it_) are important properties of low-power devices, and *J* and *D*_it_ should be less than 1.5 × 10^−2^ A cm^−^^2^ and 10^10^ cm^−2^ eV^−1^, respectively, to meet the IRDS requirements for low-power devices^3,5,32^. However, it is challenging to meet these stringent requirements for 2D devices because the amorphous oxide cannot provide a well-defined interface^5,32^, whereas crystalline dielectric materials are easy to form an abrupt interface with 2D channel, but some of them, for example, Sb_2_O_3_ (ref. ^12^), SrTiO_3_ (ref. ^13^), h-BN (ref. ^15^), have a relatively small bandgap. It is possible to address these problems by using single-crystalline c-Al_2_O_3_ because it has an adequate bandgap of 8.8 eV compared with other high-*κ* oxides^26^. To determine the Al/c-Al_2_O_3_ gate properties, the structure is transferred onto the multilayer MoS_2_ to form the Al/c-Al_2_O_3_/MoS_2_ heterostructure (Fig. 2a). The cross-sectional HR-TEM image of the heterostructure shows a stack consisting of single-crystalline Al, c-Al_2_O_3_ and MoS_2_ layers, from top to bottom, respectively. An atomically sharp interface is observed between c-Al_2_O_3_ and MoS_2_. The magnified images of the dashed green, orange and blue boxes in Fig. 2a (right) indicate single-crystalline Al, c-Al_2_O_3_ and MoS_2_, respectively. Figure 2b shows the selected area electron diffraction patterns of the dashed green, orange and blue boxes in Fig. 2a confirming the crystallinity of each stacked region. The line profiles (Fig. 2b, bottom) along the dashed green, orange and blue lines in Fig. 2a show periodic variability in the interatomic distance. The distances for Al, c-Al_2_O_3_ and MoS_2_ are derived to be 0.21 nm, 0.37 nm and 0.6 nm, respectively.

Two different types of Al_2_O_3_ dielectric materials, amorphous and crystalline, are transferred onto the MoS_2_ channel by the same integration method. The HR-TEM images of a-Al_2_O_3_/MoS_2_ are shown in Fig. 2c (left) and those of c-Al_2_O_3_/MoS_2_ are shown in Fig. 2d (left). The important tunnelling contributions of the a-Al_2_O_3_/MoS_2_ junctions are shown in Fig. 2c (right) and those of the c-Al_2_O_3_/MoS_2_ junctions are shown in Fig. 2d (right). Apart from direct tunnelling through a-Al_2_O_3_, trap-assisted tunnelling, which is observed in insulators containing a substantial number of defects, plays an important part. Conversely, these tunnelling currents are suppressed because c-Al_2_O_3_ has a wider bandgap and a smaller defect density. Figure 2e shows the experimental *J* values of c-Al_2_O_3_/MoS_2_ and a-Al_2_O_3_/MoS_2_, and c-Al_2_O_3_/MoS_2_ shows a *J* value that is about two orders of magnitude lower than that of a-Al_2_O_3_/MoS_2_.

**Extended Data Fig. 6:**
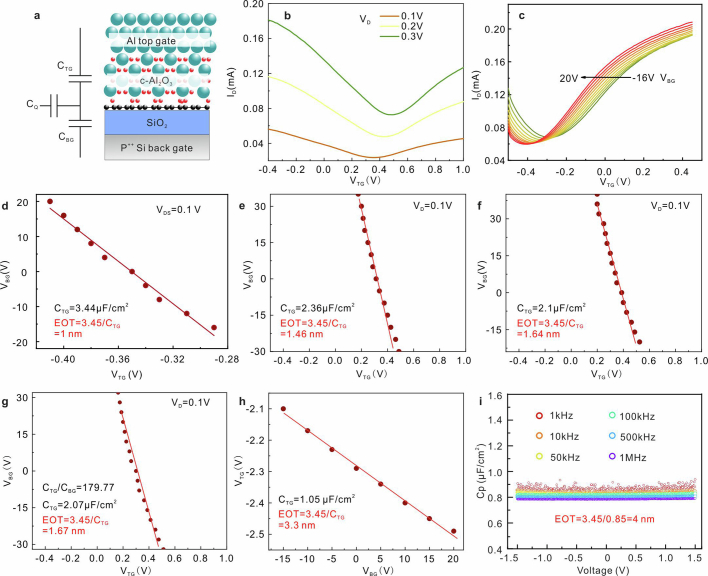
Double-gate measurement to extract EOT. a, A schematic of the double-gate graphene transistor model. *C*_*TG*_ is the top gate capacitance of c-Al_2_O_3_, *C*_*BG*_ (0.0115 μF cm^−2^) is the back gate capacitance of 300 nm SiO_2_, *C*_*Q*_ is the quantum capacitance of graphene. b, and c, Transfer curves of a typical top gate graphene transistor with c-Al_2_O_3_ as gate dielectrics and corresponding transfer curves under different *V*_*BG*_, *V*_*D*_ = 0.1 V. d-g, Dirac point voltage (*V*_TG, Dirac_) versus *V*_*BG*_ in top gate graphene transistors. From the linear fitting, EOT of 1 nm, 1.46 nm, 1.64 nm, and 1.67 nm are obtained respectively. h, Liner fitting of threshold voltage of a top gate MoS_2_ transistor, EOT of 3.3 nm is obtained. i, C-V characteristics for MIM devices with 10 nm c-Al_2_O_3_ under different frequencies, EOT of 4 nm is obtained.

A double-gate device with SiO_2_/Si as the global back gate and Al/c-Al_2_O_3_ as the top-gate stack is fabricated to measure the equivalent oxide thickness (EOT) accurately. The top-gate capacitance (*C*_TG_) is extracted experimentally as shown in Extended Data Fig. 6. The EOT is calculated to be 1.4 nm (Fig. 2f) using the equation EOT = 3.45/*C*_TG_ (ref. ^32^). Furthermore, six devices with different EOTs are fabricated as shown in Extended Data Fig. 6. Figure 2g compares the EOT values for different dielectric thicknesses (*t*_d_). The comparison of the dielectric constant with *t*_d_ is shown in Supplementary Fig. 2. As expected, EOT increases linearly with *t*_d_ for both a-Al_2_O_3_ (refs. ^35–39^) and c-Al_2_O_3_, which is consistent with the equation EOT = 3.9*t*_d_/*ε*_d_, where *ε*_d_ is the dielectric constant of Al_2_O_3_ (refs. ^30,40^). Specifically, c-Al_2_O_3_ shows the smallest EOT compared with other 2D devices based on Al_2_O_3_ reported so far and also demonstrates the potential for scaling down 2D FETs.

The *J* values of other typical amorphous oxide dielectrics^20,21,32,41–44^, c-Al_2_O_3_ dielectrics and crystalline dielectrics^11,13,15^ with varying EOTs are provided in Fig. 2h. Except for hBN, the crystalline dielectric materials with a well-defined interface have a smaller *J* than conventional amorphous oxides. As a result of the small bandgap and low permittivity, 2D hBN exhibits extremely high *J* values^15^. The c-Al_2_O_3_/MoS_2_ device shows a *J* value of 10^−6^ A cm^−2^, which is four orders of magnitude lower than the low-power requirement (10^−2^ A cm^−2^) by IRDS^3,32^. Moreover, the *J* values (EOT = 0.77 nm; Supplementary Fig. 3) are steady even when the temperature increases to about 403 K (Supplementary Fig. 4), indicating that the trap-assisted tunnelling phenomenon in amorphous dielectric materials is hardly seen in c-Al_2_O_3_. Another comparison between c-Al_2_O_3_/MoS_2_ and the mature Si-CMOS technology shows that *J* is five orders of magnitude lower than that of Al_2_O_3_/Si (ref. ^20^), suggesting great scaling-down potential for the c-Al_2_O_3_/MoS_2_ FETs.

The interface state density (*D*_it_*)* is determined by the 1/*f* noise method^30^ as shown in Fig. 2i. Because of the well-defined interface between c-Al_2_O_3_ and MoS_2_, a reduced *D*_it_ of 8.4 × 10^9^ cm^−2^ eV^−1^ is achieved, similar to the value measured by the capacitance–voltage method (Supplementary Fig. 5). The calculation details are described in the Methods. The comparison of the *D*_it_ values with those in the literature is presented in Fig. 2j, and the details are shown in Supplementary Table 3. Most of the *D*_it_ values of exfoliated MoS_2_ channels are high ranging from 5 × 10^11^ cm^−2^ eV^−1^ to 10^13^ cm^−2^ eV^−1^ for both conventional amorphous oxide dielectrics^31,32,37,43–49^ and crystalline dielectrics^11,13,15^. In our case, a *D*_it_ of 8.4 × 10^9^ cm^−2^ eV^−1^ is achieved from c-Al_2_O_3_/MoS_2_, and it meets the IRDS requirement for low-standby-power CMOS and is comparable to that of Si/SiO_2_ (*D*_it_ ~ 10^9^ cm^−2^ eV^−1^) (ref. ^50^).

**Fig. 3:**
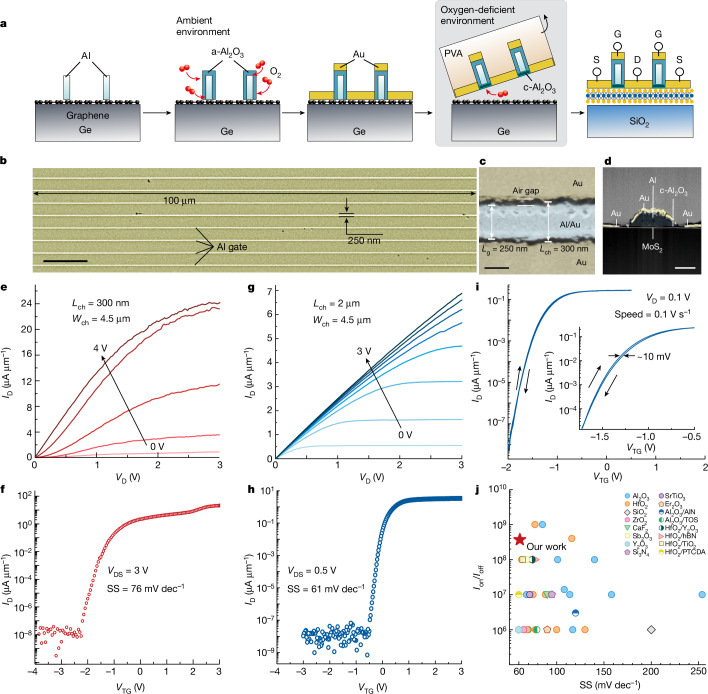
Fabrication and electronic characteristics of c-Al_2_O_3_/MoS_2_ FET. **a**, The fabrication process of a self-aligned c-Al_2_O_3_/MoS_2_ FET. **b**, SEM image of an array of self-aligned MoS_2_ FETs. **c**, Magnified SEM image of self-aligned MoS_2_ FET with a small air gap. **d**, Cross-sectional TEM image of self-aligned MoS_2_ FET. **e**,**f**, The output (**e**) and transfer (**f**) characteristics of a short-channel three-layered MoS_2_ FET. **g**,**h**, The output (**g**) and transfer (**h**) characteristics of a long-channel three-layered MoS_2_ FET. **i**, The dual-sweep transfer curve. **j**, Experimental SS values versus *I*_on_/*I*_off_ of MoS_2_ FETs with various dielectrics. Scale bars, 10 μm (**b**); 200 nm (**c**); 100 nm (**d**). Source data

To study the electronic characteristics of the 2D FET based on c-Al_2_O_3_, a self-aligned MoS_2_ FET with 2 nm c-Al_2_O_3_ is fabricated by the vdW transfer method. The fabrication process of the self-aligned MoS_2_ FET is shown in Fig. 3a and Supplementary Fig. 6. Figure 3b shows the scanning electron microscopy (SEM) image of an array of the self-aligned MoS_2_ FETs. The width and length of the Al gate are 100 μm and 250 nm, respectively. A small air gap between Au and the Al gates, which ensures the complete insulation between the top gate and source or drain and the successful self-alignment process, is shown in Fig. 3c. The channel length is 300 nm (*L*_ch_ = 300 nm). The cross-sectional TEM image of self-aligned MoS_2_ FET is shown in Fig. 3d. The vdW transfer method enables the fabrication of the complete FET stack by the self-alignment process, including the source, drain, dielectric and gate on the graphene/Ge donor wafer, and then it is transferred onto the channel materials in a one-step lamination process to produce the 2D FET with a good contact and dielectric interfaces^48^.

**Extended Data Fig. 7:**
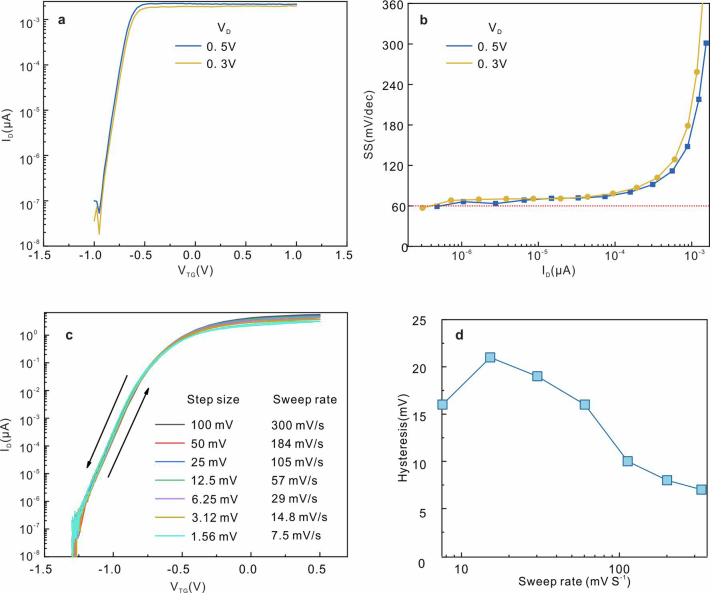
SS distribution and hysteresis of vdW-transferred c-Al_2_O_3_ top-gate MoS_2_ transistors. a, Transfer curves of top-gate MoS_2_ transistor. b, SS distribution as a function of channel current in a. The dashed line represents the thermal limit of SS at room temperature (60 mV/dec). c, The double-sweep transfer curves with different step size and sweeping rates of MoS_2_ transistor using the transferred c-Al_2_O_3_/Al gate stack. d, Hysteresis versus sweep rate.

**Extended Data Fig. 8:**
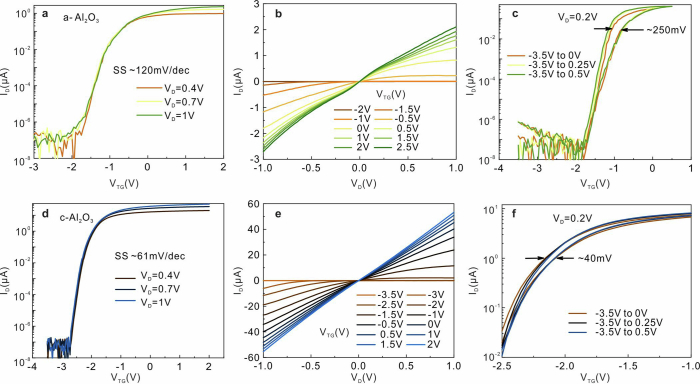
Electrical performance of a-Al_2_O_3_ and c-Al_2_O_3_ top-gate MoS_2_ transistors. a, and b, Transfer and output curves of ALD a-Al_2_O_3_ top-gate MoS_2_ transistors. c, The double-sweep transfer curves with different gate-voltage ranges of ALD a-Al_2_O_3_ top-gate MoS_2_ transistors. d, and e, Transfer and output curves of vdW-transferred c-Al_2_O_3_ top-gate MoS_2_ transistors. f, Enlarged double-sweep transfer curves with different gate-voltage ranges of vdW-transferred c-Al_2_O_3_ top-gate MoS_2_ transistors. The top-gate MoS_2_ transistors with vdW-transferred c-Al_2_O_3_ dielectric and Au contracts show a higher on/off ratio (1.05 × 10^8^), smaller subthreshold swing (*SS*  ≈  61 mV/dec) and hysteresis (40 mV). By contrast, the devices with ALD a-Al_2_O_3_ and deposited Au contacts show a notably smaller on/off ratio (1.4 × 10^7^), larger *SS* (120 mV/dec), and hysteresis (250 mV).

The output (drain current versus drain voltage, *I*_D_*–V*_D_) and transfer (drain current versus gate voltage, *I*_D_–*V*_TG_) characteristics of the MoS_2_ FET are shown in Fig. 3e,f. The output characteristics demonstrate promising current control and saturation. The drain current of the transfer curves shows a steep increase in the subthreshold region with a subthreshold swing (SS) of about 76 mV dec^−1^ and an on/off current ratio (*I*_on_*/I*_off_) of 10^9^. The output and transfer characteristics of the long-channel MoS_2_ FET are shown in Fig. 3g,h. A small SS of 61 mV dec^−1^ (Extended Data Fig. 7) close to the thermal limit of 60 mV dec^−1^ at 300 K is accomplished. A small hysteresis of 10 mV is also observed in the dual-sweep linear transfer curve in Fig. 3i, indicating a low trapping charge density in c-Al_2_O_3_. Moreover, a contrast experiment is performed using FETs containing c-Al_2_O_3_ and a-Al_2_O_3_ (Extended Data Fig. 8). The SS and hysteresis obtained from the device with c-Al_2_O_3_ (SS = 61 mV dec^−1^ and hysteresis of 40 mV) are notably lower than those of a-Al_2_O_3_ (SS = 120 mV dec^−1^ and hysteresis of 250 mV). Figure 3j shows the SS versus *I*_on_/*I*_off_ results of MoS_2_ FETs comprising different dielectrics^10–13,27,30–33,37,42,44,46–49^, and the details are shown in Supplementary Table 4. Specifically, the MoS_2_ FET using c-Al_2_O_3_ as gate dielectric delivers the best overall electrical performance such as low *SS* and high *I*_on_/*I*_off_.

**Fig. 4:**
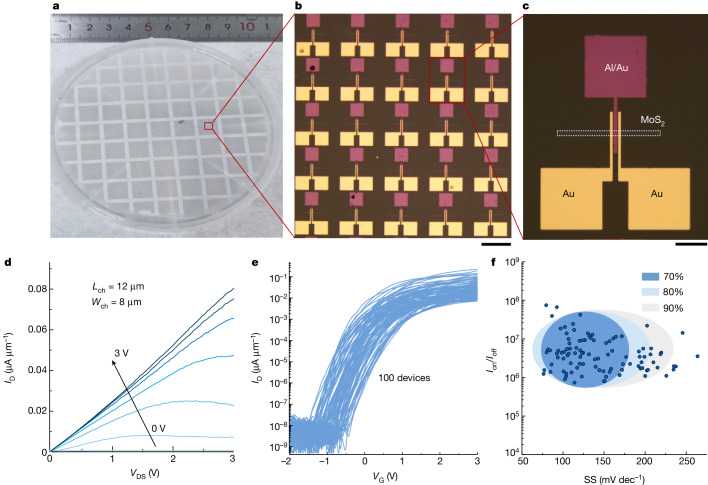
Batch-fabricated c-Al_2_O_3_/MoS_2_ FETs on a 4-inch CVD-MoS_2_/sapphire wafer. **a**, Photograph of a 4-inch CVD-MoS_2_/sapphire wafer with top-gate FET arrays. **b**, Magnified optical image from the red box in **a**. **c**, Magnified optical image from the red box in **b**. **d**, The typical output curves of c-Al_2_O_3_/MoS_2_ FETs. **e**, Transfer curves of 100 MoS_2_ FETs. **f**, The statistic distributions of the on/off current ratio and SS from 100 devices. Scale bars, 200 μm (**b**); 50 μm (**c**). Source data

**Extended Data Fig. 9:**
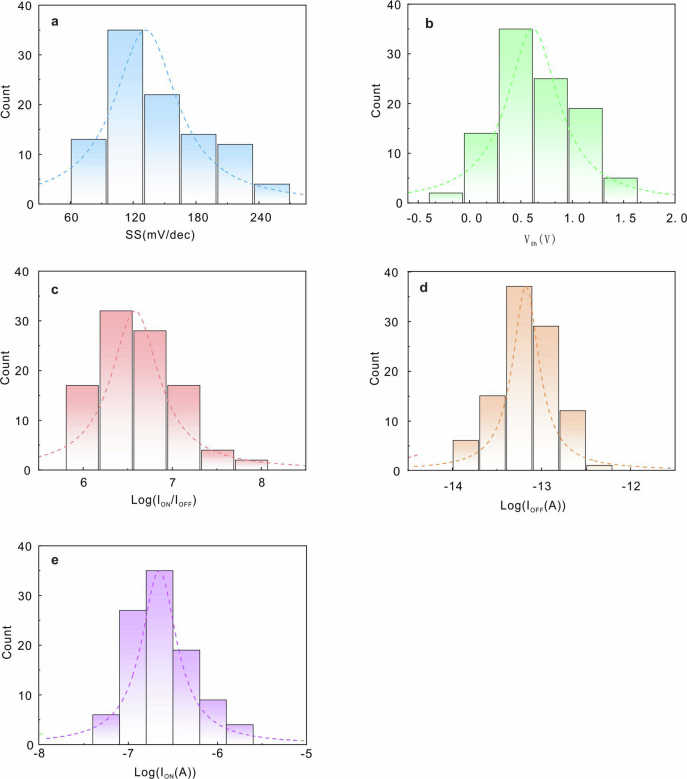
Electrical performance of CVD-MoS_2_ transistor array. Histogram and Lorentz’s distribution fits of a, subthreshold swing SS, b, threshold voltage, *V*_*th*_, c, on/off ratio *I*_*ON*_/*I*_*OFF*_, d, off-state current *I*_*OFF*,_ and e, on-state current *I*_*ON*_ extracted from the transfer curves of 100 devices. According to Lorentz distribution fittings, the statistical results of *SS*, *V*_*th*_, *I*_*ON*_/*I*_*OFF*_, *I*_*OFF*_, and *I*_*ON*_ show centralized distributions with average values of 121.8 mV/dec, 0.63 V, 3.2 × 10^6^, 7 × 10^−14 ^A, 2 × 10^−7 ^A, respectively. The electrical performance and uniformity of MoS_2_ transistors can be further improved by using single crystalline CVD MoS_2_ wafer in the future.

**Extended Data Fig. 10:**
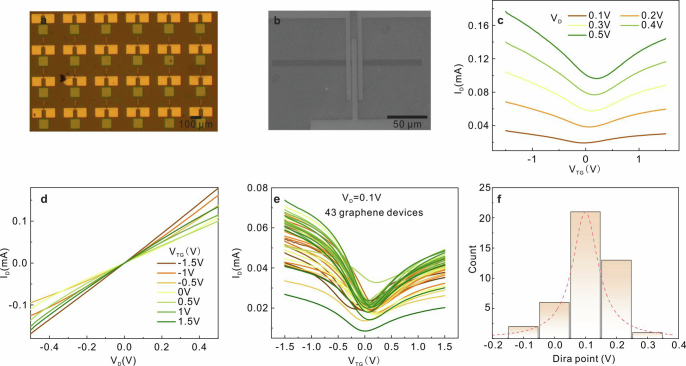
Electrical performance of CVD-grown graphene transistor array. a, Optical image of the top-gate graphene transistor array with vdW-transferred c-Al_2_O_3_ dielectric and Au contracts. b, SEM image of a single graphene transistor in a. c and d, Transfer and output curves of a typical graphene transistor. e, Transfer curves of 43 graphene transistors in the array, *V*_*D*_ = 0.1 V. f, Histogram and Lorentz’s distribution fits of Dirac point voltage, the Dirac point voltage shows centralized distribution with average values of 0.13 V.

To further demonstrate the scalable fabrication of large-area top-gate FETs, the FET arrays are prepared on a 4-inch chemical vapour deposition(CVD)-MoS_2_ wafer. The photograph of the 4-inch CVD-MoS_2_/sapphire wafer with the top-gate FET arrays in Fig. 4a confirms that the complete FET stack is transferred to the MoS_2_/sapphire substrate. Figure 4b,c does not show any wrinkles or cracks. The typical output curves of these transistors demonstrate excellent electrostatic control of the channel because of the high-quality dielectrics (Fig. 4d). The transfer curves of 100 MoS_2_ FETs exhibit the typical n-type characteristics with excellent uniformity (Fig. 4e). The statistical distributions of the on/off current ratio (*I*_on_/*I*_off_) and SS extracted from 100 devices are shown in Fig. 4f and further statistical distributions of the key parameters, including the threshold voltage, off-state current and on-state current are shown in Extended Data Fig. 9. Seventy percent of the devices show SS values in the range of 75–175 mV dec^−1^ and *I*_on_/*I*_off_ higher than 10^6^, which are among the best for CVD-MoS_2_ FET. The value and distribution of SS for MoS_2_ transistor array can be markedly improved as wafer-scale single-crystalline MoS_2_ with excellent quality is available and used in the future. The uniformity and scalability of the c-Al_2_O_3_/graphene FETs shown in Extended Data Fig. 10 indicate the ability of the fabricating high-performance radio frequency transistors and other complex circuits with c-Al_2_O_3_ technique in the future.

In conclusion, we have demonstrated the fabrication of single-crystalline Al_2_O_3_ as the high-quality dielectric layer for the top-gate 2D transistors. This breakthrough will serve as the foundation for further advancements in the diversity, scalability and manufacturability of single-crystalline oxides, facilitating the seamless transition of 2D semiconductors from laboratories to industrial environments. The approach for the growth of wafer-scale single-crystalline aluminium and aluminium oxide can be further extended to other metals, and some single-crystalline oxides that were unattainable before can be synthesized for various applications. A notable advancement in this journey is the ability to scale up to the current silicon wafer diameter because the starting germanium for single-crystalline graphene synthesis can be epitaxially grown on silicon directly, which markedly broadens the potential for integration into established silicon-factory manufacturing processes. Meanwhile, the development of an automated debonding-transfer tool dedicated to low-dimensional materials, including single-crystalline dielectric oxide, 2D channel material and ultrathin metal electrode, is crucial to achieve the unique alignments between them for multifunctional 2D devices and is expected to substantially increase throughput, improve reproducibility and enhance the reliability of 2D integrated circuits. By constructing complex 2D integrated circuits, particularly for hetero-integration on mature Si-CMOS platforms, the immense potential of 2D materials can be fully unlocked and can lay the groundwork for the next generation of high-performance electronic devices.

## Methods

### Synthesis of single-crystalline Gr/Ge(110) template substrate

A p-type Ge(110) wafer was placed in the quartz tube in a CVD system evacuated to 0.1 Pa. After switching off the vacuum pump, the quartz tube was heated to 916 °C under a flow of H_2_ (30 sccm) and Ar (300 sccm). When the temperature of the quartz tube reached 916 °C, Gr growth proceeded under a flowing gas mixture of CH_4_ (0.8 sccm), H_2_ (70 sccm) and Ar (700 sccm) for 600 min. Then, CH_4_ and H_2_ were turned off, and the quartz tube cooled to room temperature under Ar (200 sccm). In this process, single-crystalline Gr/Ge(110) template substrate was synthesized successfully^25^.

### Materials preparation

In the fabrication of a single MoS_2_ transistor, the MoS_2_ flakes were exfoliated mechanically and were dry-transferred to a 300-nm SiO_2_/p++ silicon substrate using the PF gel films (Gel Pak). In the batch fabrication of MoS_2_ transistors, the wafer-scale polycrystal monolayer CVD-grown MoS_2_ on sapphire was patterned into arrays by RIE (50 sccm O_2_, 30 W and 10 Pa) and photolithography. In the batch fabrication of graphene transistors, the CVD Gr grown on the Ge substrate was transferred to 300 nm SiO_2_/p++ silicon by PMMA-assisted transfer and patterned into arrays by RIE (50 sccm O_2_, 30 W and 10 Pa) and photolithography. All the 2D materials were vacuum annealed at 250 °C for 2 h to remove the photoresist and absorbents.

### Device fabrication

An Al film with a thickness of 100 nm was deposited by an electron beam evaporation at 10^−7^ torr at a rate of 0.6 Å s^−1^. Electron beam lithography and photolithography and electron beam evaporation (Texas Instruments DE400) were used to define the Al nanoribbon electrodes on the Gr/Ge(110) substrate after the lift-off process. The top and side of Al nanoribbons were oxidized to form a-Al_2_O_3_ in an ambient environment. Afterwards, 10–15 nm thick Au was deposited on the Gr/Ge(110) template to form the self-aligned source (S)/drain (D) at a rate of 0.1 Å s^−1^. Then, the dry polyvinyl alcohol (PVA) film was laminated on the self-aligned structure at 90 °C for 3 min by a PDMS stamp. In an anaerobic glovebox (0.2 ppm oxygen), the self-aligned device stack together with PVA was peeled off and physically laminated on top of the mechanically exfoliated few-layer MoS_2_ (or wafer-scale CVD-MoS_2_ on sapphire) at 90 °C for 3 min by a PDMS stamp using a microscopic alignment system. After the transfer process, c-Al_2_O_3_ was formed as the gate dielectric on Al in an oxygen-deficient environment (0.2 ppm O_2_). Finally, the PVA film was dissolved in dimethyl sulfoxide (DMSO). For the metal–insulator–metal (MIM) capacitor, the Cr/Au (10/50 nm) bottom electrodes were deposited on the sapphire substrate to eliminate the parasitic capacitance.

### Cross-sectional TEM sample preparation and characterization

The patterned Au/Al bilayer with a thickness of 20 nm/100 nm was deposited on the Gr/Ge(110) substrate by electron beam evaporation. The dry PVA film was laminated on top of the Au/Al patterns at 90 °C for 3 min by a PDMS stamp. The Au/Al patterns together with PVA were peeled off from the Gr/Ge(110) substrate and attached to a PDMS stamp. To change the thickness of c-Al_2_O_3_, the Au/Al patterns transferred onto PVA were exposed to an oxygen-deficient environment for different durations after the peeling process. Finally, the Au/Al/c-Al_2_O_3_ patterns were transferred on the MoS_2_, SiO_2_ and sapphire substrates at 90 °C for 3 min by a PDMS stamp on a microscopic alignment system. The PVA film was dissolved in dimethyl sulfoxide (DMSO). The entire transfer process was carried out in an anaerobic glovebox (0.2 ppm oxygen). A 20-nm Au protection layer was deposited directly on the whole substrate, which also serves as the conduction layer for the subsequent focused-ion-beam process. The cross-sectional sample was prepared by focused-ion-beam process (Helios G4 UX, Thermo Fischer) with 30 keV Ga^+^ and the sample was examined by TEM (aberration-corrected JEM-ARM300F) at 300 kV.

### Device and materials characterization

Raman scattering was performed on the HORIBA Jobin Yvon HR800 with a 514-nm excitation laser to monitor the quality of CVD Gr/Ge and MoS_2_. The surface topography of Gr/Ge was assessed by atomic force microscopy (Multimode 8, Bruker) using the tapping mode. The surface topography and structure of the devices were examined by SEM (Zeiss SUPRA 55). The electrical measurements were performed under ambient conditions at room temperature in the dark on the semiconductor parameter analyser (Agilent B1500A) and commercial high-resolution photoelectric scanner (MStarter200).

### EOT extraction

The EOT was estimated based on the linear relationship of the Dirac point voltage (*V*_TG, Dirac_) drift of the top-gate graphene transistor or the threshold voltage (*V*_TH_) drift of the top-gate MoS_2_ transistor as a function of the back-gate voltage (*V*_BG_) (ref. ^32^) by the following equation:

$$\frac{{C}_{{\rm{TG}}}}{{C}_{{\rm{BG}}}}=-\frac{\Delta {V}_{{\rm{BG}}}}{\Delta {V}_{{\rm{TG}},{\rm{Dirac}}}}{\rm{;}}\frac{{C}_{{\rm{TG}}}}{{C}_{{\rm{BG}}}}=-\frac{\Delta {V}_{{\rm{BG}}}}{\Delta {V}_{{\rm{TH}}}};\,{\rm{EOT}}=\frac{3.45\,{\rm{\mu }}{\rm{F}}\,{{\rm{cm}}}^{-2}}{{C}_{{\rm{TG}}}},$$

where 3.45 μF cm^−2^ is the gate capacitance of 1 nm SiO_2_, *C*_TG_ is the top-gate capacitance and *C*_BG_ is the back-gate capacitance of 300 nm SiO_2_.

### Interface states extraction

The interface state, *D*_it_, was extracted according to the 1/*f* noise method^30^ using the following equation:

$$\frac{{S}_{I}}{{I}_{{\rm{ds}}}^{2}}={\left(\frac{{g}_{{\rm{m}}}}{{I}_{{\rm{ds}}}}\right)}^{2}{S}_{{\rm{vfb}}};\,{S}_{{\rm{vfb}}}=\frac{{q}^{2}{K}_{{\rm{B}}}T{D}_{{\rm{it}}}}{WL{C}_{{\rm{OX}}}^{2}\,f},$$

where *S*_*I*_ is spectral density, *g*_m_ is the gate transconductance, *S*_vfb_ is the flat band voltage spectral density, *q* is the electronic charge, *K*_B_ is Boltzmann’s constant, *T* is the temperature, *W* and *L* are the width and the length of the channel, respectively, and *C*_ox_ is the gate capacitance per unit area.

The interface state, *D*_it_, was extracted according to the *C*–*V* method^48^ using the following equation:

$${D}_{{\rm{it}}}=\frac{1}{q}\left[{\left(\frac{1}{{C}_{{\rm{LF}}}}-\frac{1}{{C}_{{\rm{ox}}}}\right)}^{-1}-{\left(\frac{1}{{C}_{{\rm{HF}}}}-\frac{1}{{C}_{{\rm{ox}}}}\right)}^{-1}\right],$$

where *q* is the electronic charge, *C*_ox_ is the gate capacitance per unit area, *C*_LH_ and *C*_HF_ are the measured quasistatic (low-frequency) and high-frequency depletion capacitance per unit area, respectively.

## Online content

Any methods, additional references, Nature Portfolio reporting summaries, source data, extended data, supplementary information, acknowledgements, peer review information; details of author contributions and competing interests; and statements of data and code availability are available at 10.1038/s41586-024-07786-2.

## Supplementary information

Supplementary Information This file contains Supplementary Figs. 1–6 and Supplementary Tables 1–5.

Supplementary Video 1 Peeling process of the 4-inch patterned Al film from the Gr/Ge substrate using PVA.

## Source data

Source Data Fig. 1

Source Data Fig. 2

Source Data Fig. 3

Source Data Fig. 4

## Data Availability

Source data are provided with this paper.
