# Publisher Correction: Single-crystalline metal-oxide dielectrics for top-gate 2D transistors

**DOI:** 10.1038/s41586-024-08001-y

**Published:** 2024-09-06

**Authors:** Daobing Zeng, Ziyang Zhang, Zhongying Xue, Miao Zhang, Paul K. Chu, Yongfeng Mei, Ziao Tian, Zengfeng Di

**Affiliations:** 1 State Key Laboratory of Materials for Integrated Circuits, Shanghai Institute of Microsystem and Information Technology, Chinese Academy of Sciences, Shanghai, China; 2 Center of Materials Science and Optoelectronics Engineering, University of Chinese Academy of Sciences, Beijing, China; 3 Department of Physics, Department of Materials Science and Engineering, and Department of Biomedical Engineering, City University of Hong Kong, Kowloon, China; 4 Department of Materials Science, Fudan University, Shanghai, China

**Keywords:** Electronic devices, Two-dimensional materials

Correction to: *Nature* 10.1038/s41586-024-07786-2 Published online 7 August 2024

In the version of the article initially published, in the key to Fig. 1g, the label now reading “c-Al_2_O_3_ 2 nm” originally read “a-Al_2_O_3_ 2 nm” and has now been amended in the HTML and PDF versions of the article.

**Fig. 1:**
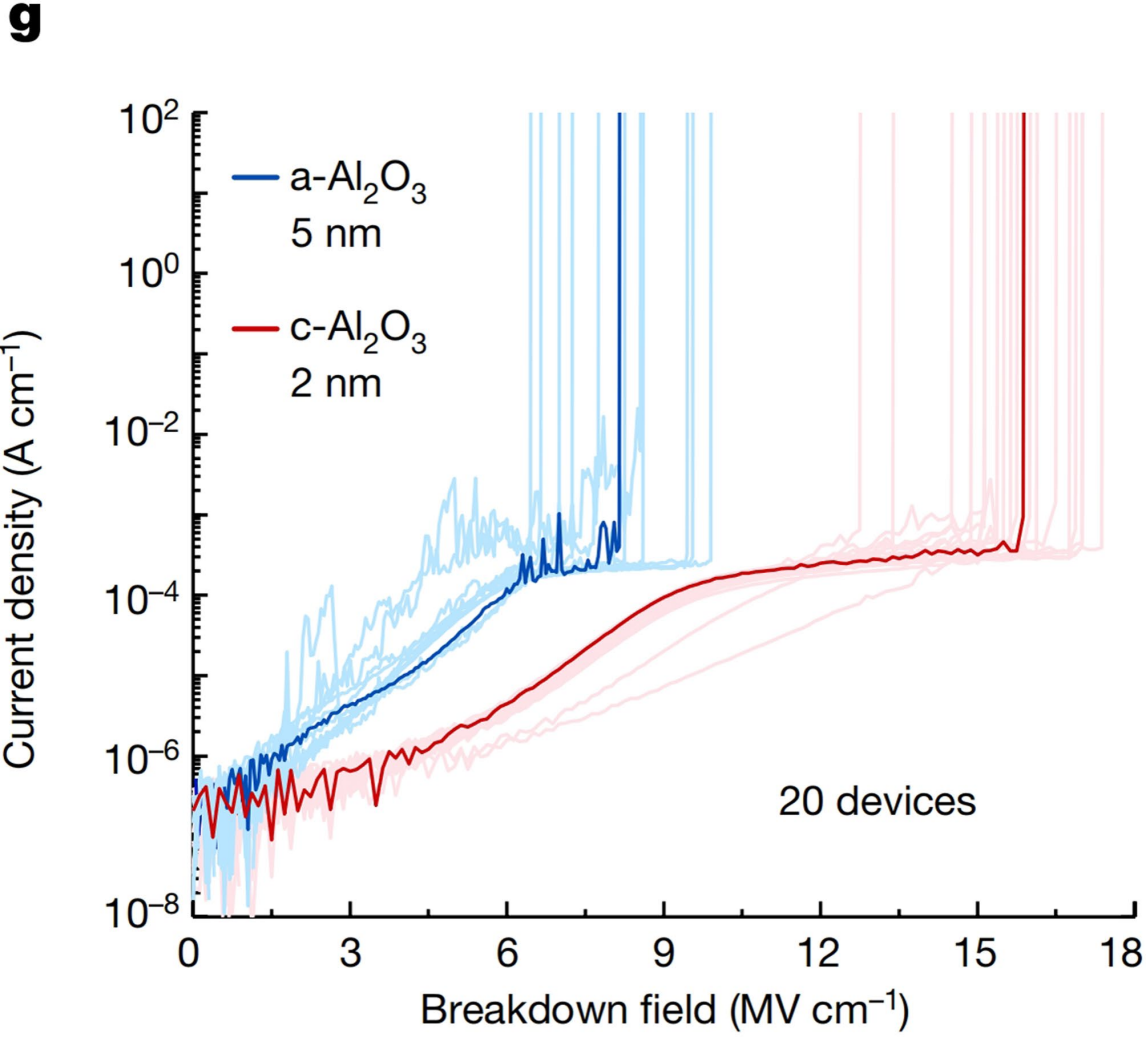
Correct figure .

