# Supplementary material for: Single-crystalline metal-oxide dielectrics for top-gate 2D transistors
Source: Nature. 2024 Aug 7;632(8026):788–94. doi: 10.1038/s41586-024-07786-2 (PMC11338823; doi:10.1038/s41586-024-07786-2)
Supplement: Supplementary file 1 — This file contains Supplementary Figs. 1–6 and Supplementary Tables 1–5. [file 41586_2024_7786_MOESM1_ESM.pdf]

nature portfolio

https://doi.org/10.1038/s41586-024-07786-2

Supplementary information

Single-crystalline metal-oxide dielectrics for top-gate 2D transistors

In the format provided by the authors and unedited

Supplementary Information for

Single-crystalline metal-oxide dielectrics for top-gate 2D transistors

Daobing Zeng[1,2], Ziyang Zhang[1,2], Zhongying Xue[1], Miao Zhang[1], Paul K. Chu[3], Yongfeng Mei[4], Ziao Tian[1*], & Zengfeng Di[1*]

[1] State Key Laboratory of Materials for Integrated Circuits, Shanghai Institute of Microsystem and Information Technology, Chinese Academy of Sciences, Shanghai 200050, China.

[2] Center of Materials Science and Optoelectronics Engineering, University of Chinese Academy of Sciences, Beijing 100049, China.

[3] Department of Physics, Department of Materials Science and Engineering, and Department of Biomedical Engineering, City University of Hong Kong, Tat Chee Avenue, Kowloon, Hong Kong, China.

[4] Department of Materials Science, Fudan University, Shanghai 200433, China.

* email: zatian@mail.sim.ac.cn (Z.A. Tian); zfdi@mail.sim.ac.cn (Z.F. Di)

1. The thickness mapping of 4-inch c-Al_2O_3/Al wafer.

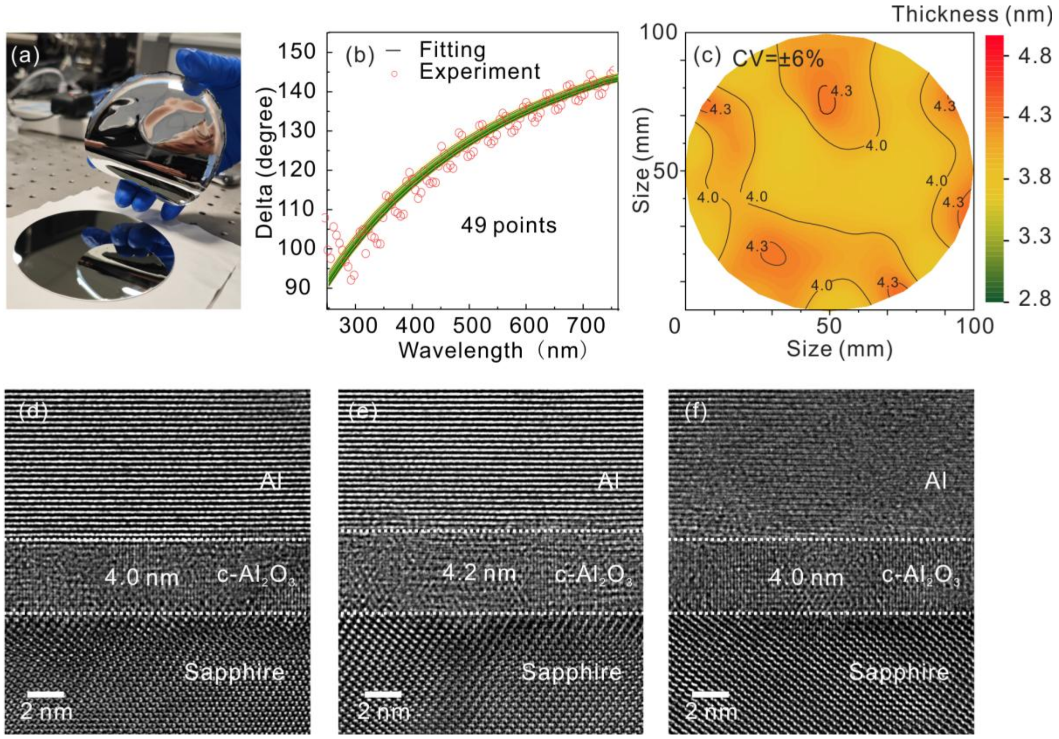

Supplementary Figure 1. The thickness mapping of 4-inch c-Al_2O_3/Al wafer. (a) 4-inch c-Al_2O_3/Al wafer peeling from graphene/Ge(110) substrate. (b) The thicknesses fitting of 4-inch c-Al_2O_3/Al wafer with 49 data measured by ellipsomete. (c) Thickness mapping. The thickness values of c-Al_2O_3 range from 3.7 nm to 4.3 nm, and the average thickness for the 49 points is 4.0 nm. The standard deviation is 0.046 nm. The coefficient of variation (CV) is ± 6%. (d)-(f) Randomly selected cross-sectional HRTEM images at three different locations on the 4-inch c-Al_2O_3/Al wafer.

2. Dielectric constants of c-Al_2O_3 and a-Al_2O_3.

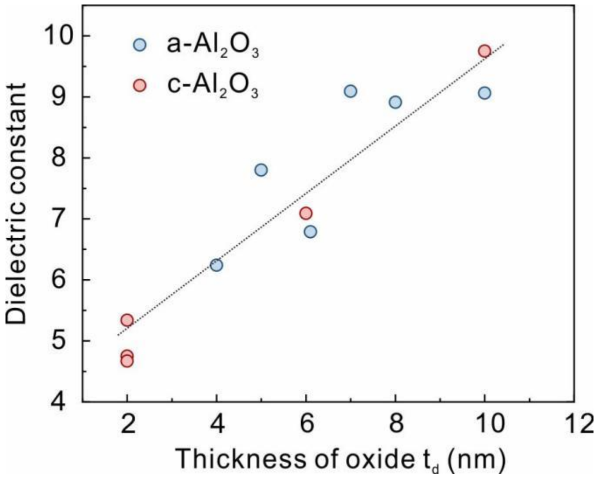

Supplementary Figure 2. Dielectric constants of c-Al_2O_3 and a-Al_2O_3. Dielectric constants of c-Al_2O_3 and a-Al_2O_3 with variable oxide thickness.

3. The equivalent oxide thickness (EOT) of c-Al_2O_3

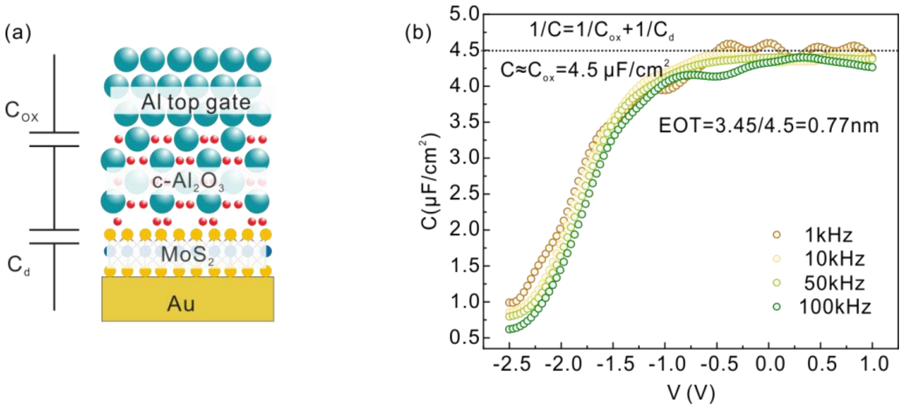

Supplementary Figure 3. The equivalent oxide thickness (EOT) of c-Al_2O_3. (a) The equivalent circuit model and vertical Metal-Insulator-Semiconductor (MOS) capacitors (Al/c-Al_2O_3/MoS_2/Au). (b) The capacitance-voltage (C-V) of the vertical MOS capacitor under different frequencies. The EOT is calculated by EOT=3.45 μF cm^{-2}/C_{ox}, where 3.45 μFcm^{-2} is the gate capacitance for 1nm SiO_2. EOT=0.77 nm.

4. The mechanisms underlying the low leakage current of c-Al_2O_3

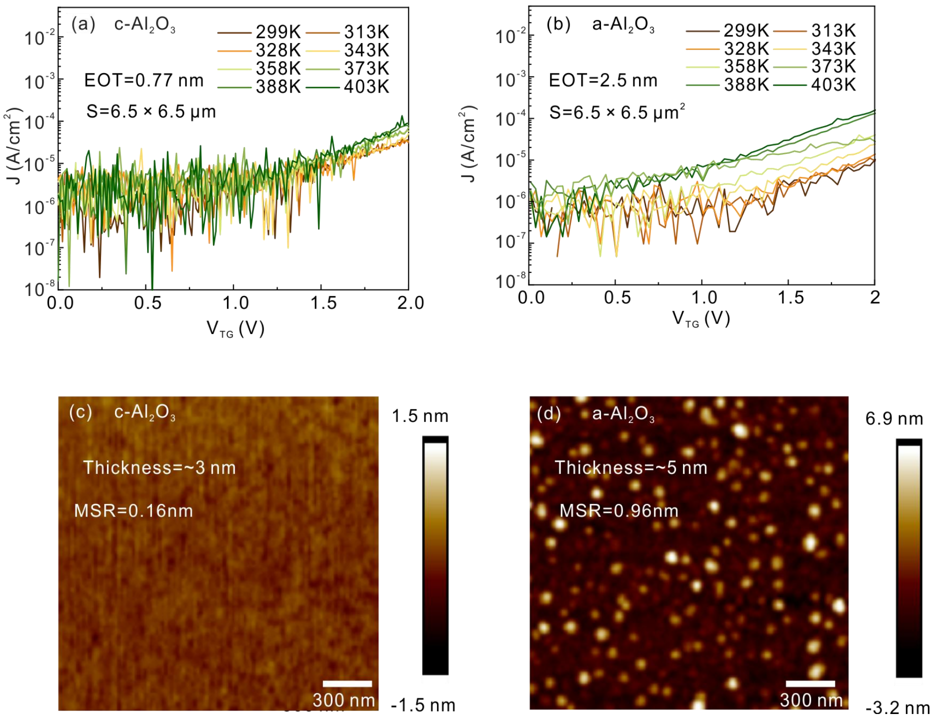

Supplementary Figure 4. The mechanisms underlying the low leakage current of c-Al_2O_3. The temperature dependence of the leakage current of MOS capacitor (Al/ Al_2O_3/MoS_2/Au) based on (a) c-Al_2O_3 and (b) a-Al_2O_3 at low gate voltages. The area of MOS capacitor is 6.5×6.5μm. AFM images of (c) 3-nm-thick c-Al_2O_3 surface and (d) 5-nm-thick a-Al_2O_3 surface.

The significantly lower leakage current in c-Al_2O_3 compared to a-Al_2O_3 and other dielectrics is attributed to the suppression of the trap-assisted tunneling (TAT) phenomenon. In Supplementary Fig. 4 (a) and (b), the a-Al_2O_3 exhibits an EOT of 2.5 nm, larger than the 0.77 nm EOT of c-Al_2O_3. While, the leakage current of a-Al_2O_3 is comparable to that of c-Al_2O_3. Moreover, our results show that the leakage current in c-Al_2O_3 remains stable with temperature variations, unlike a-Al_2O_3, where its leakage current increases with the increment of temperature. The observed difference suggests the TAT in c-Al_2O_3 is greatly suppressed while it still exists in a-Al_2O_3.

In addition to the suppression of TAT, the atomically smooth surface of c-Al_2O_3 may be another crucial factor determining the obtained low leakage current. As reported by Timp et al.[1], the leakage current is highly dependent on the root-mean-square (RMS) roughness of the oxide, and it can even increase tenfold for every 0.1-nm increment in RMS through a 1-nm thick oxide. Supplementary Fig. 4 (c) and (d)

show that the c-Al_2O_3 surface has an RMS of 0.16 nm, which is significantly smoother than the 0.96 nm RMS of a-Al_2O_3, therefore, the leakage current of c-Al_2O_3 is expected to be much lower than that of a-Al_2O_3.

Meanwhile, the large conduction band offset ($\Delta\Phi_{CB}$) between c-Al_2O_3 and MoS_2 also plays a crucial role in reducing leakage current. Supplementary Table 5 provides the $\Delta\Phi_{CB}$ values between MoS_2 and various candidate gate dielectrics. It is shown c-Al_2O_3 possesses the second highest $\Delta\Phi_{CB}$, is only surpassed by SiO_2, while is higher than that of a-Al_2O_3 and other dielectrics. The pronounced $\Delta\Phi_{CB}$ effectively limits the injection of electrons into the oxide bands, leading to a reduction in leakage current.

5. C-V of the MoS_2/c-Al_2O_3

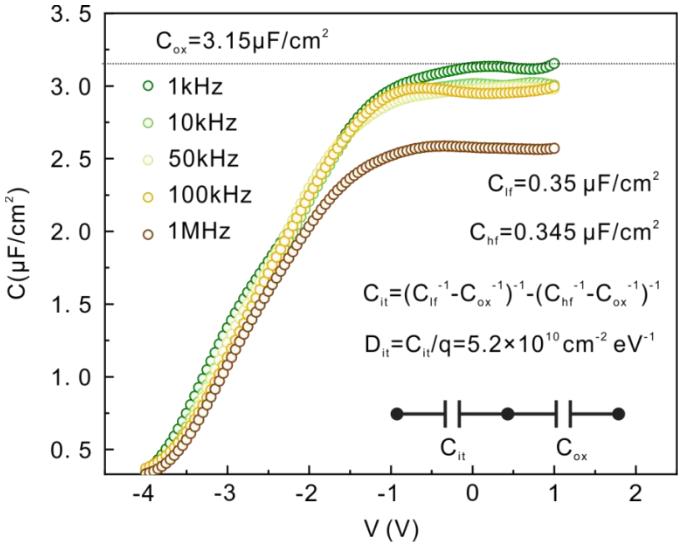

Supplementary Figure 5. C-V of the MoS_2/c-Al_2O_3. Inset illustrates the equivalent circuit model. C_{ox} is the oxide capacitance; C_{it} is the capacitance of the traps. At depletion state V= -4 V, C_{it} can be extracted from the formula: $C_{it} = (C_{lf}^{-1}-C_{ox}^{-1})^{-1}-(C_{hf}^{-1}-C_{ox}^{-1})^{-1}$, C_{lf} and C_{hf} are the the measured quasistatic (low frequency) and high frequency depletion capacitance, respectively. $D_{it} = 5.2\times10^{10}$ $cm^{-2}eV^{-1}$.

6. The fabrication process of the self-aligned MoS_2 FET

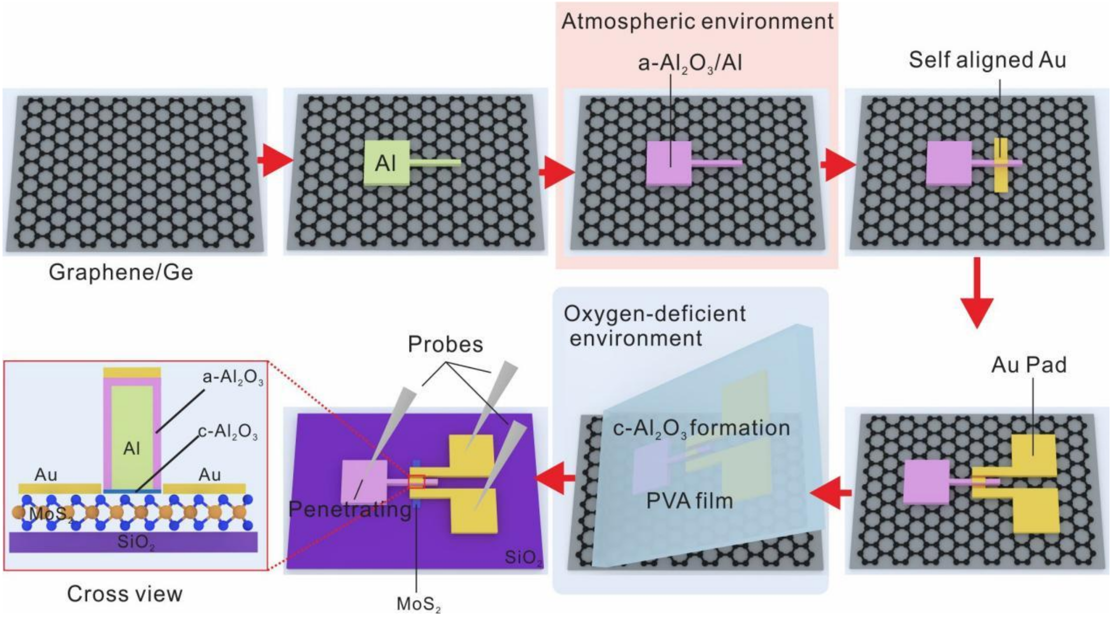

Supplementary Figure 6. The fabrication process of the self-aligned MoS_2 FET. The schematic fabrication process of the self-aligned MoS_2 FET using the transferred c-Al_2O_3/Al gate stack.

Supplementary Table 1. The thicknesses of c-Al_2O_3 at 49 different locations on the 4-inch c-Al_2O_3/Al wafer

Number	#1	#2	#3	#4	#5	#6	#7	#8	#9	#10
Thickness(nm)	3.86	4.07	3.61	3.7	3.96	3.95	3.67	4.14	4.31	4.05
Number	#11	#12	#13	#14	#15	#16	#17	#18	#19	#20
Thickness(nm)	4.31	4.21	4.27	3.86	3.84	4.19	4.01	3.9	4.12	3.77
Number	#21	#22	#23	#24	#25	#26	#27	#28	#29	#30
Thickness(nm)	4.15	4.29	4.13	4.21	4.14	3.7	3.55	3.66	3.55	3.63
Number	#31	#32	#33	#34	#35	#36	#37	#38	#39	#40
Thickness(nm)	4.11	3.74	4.18	4.2	4.17	3.92	3.94	4.37	3.73	4.06
Number	#41	#42	#43	#44	#45	#46	#47	#48	#49	
Thickness(nm)	4.41	4.08	3.85	4.45	4.01	4.44	4.18	4.17	4.13	

Supplementary Table 2. Comparison of the breakdown field versus film thickness for various metal oxides.

Dielectrics	Thickness (nm)	Breakdown fields (MV/cm)	References
Er_2O_3	7.8	7.1	1
HfO_2/Y_2O_3	28/4	4.8	2
	9/4	7	
TiO_2	9.5	2	3
	15.4	1.9	
	23	1.8	
	31	2.5	
HfO_2	3	9.33	4
	6.5	9.2	
	10	7.6	
	12	6.9	
	15	5.8	
HfO_2/TiO_2	4/1.2	10	5
	8/1.2	7.3	
	16/1.2	5.6	
Al_2O_3	4.5	12	6
	8.3	8.1	
	11.5	7.5	
	22	6.5	
HfO_2/PTCDA	1.45	16.5	
	3	12	

	6	9.4	7
	10.5	8.1	
	15.6	7.5	
$SrTiO_3$	8	5.03	8
	16	5.75	
	32	6.04	
Sb_2O_3	10	2.9	9
	20	1.8	
CaF_2	2.5	27.8	40
c-Al_2O_3	2	17.4	This work

Supplementary Table 3. Comparison of the interface state density (D_{it}) versus EOT for various gate dielectrics.

Gates	Dielectrics	Channels	EOT (nm)	D_{it} ($cm^{-2}eV^{-1}$)	Refs.
Back gate	30 nm HfO_2	exfoliated MoS_2	6.2	2.3×10^{12}	10
	15 nm Al_2O_3	exfoliated MoS_2	8	6.6×10^{12}	11
	8 nm BN	exfoliated MoS_2	7.8	5.2×10^{9}	12
	1.2 nm TiO_2/16 nm HfO_2	CVD MoS_2	4.1	1.74×10^{12}	5
	2 nm CaF_2	CVD MoS_2	0.9	1.2×10^{13}	13
	10 nm HfO_2	exfoliated MoS_2	4.25	5×10^{11}	14
	4 nm HfO_2	MOCVD MoS_2	1.9	3.9×10^{12}	15
	8 nm HfO_2		2.7	8×10^{12}	
	12 nm HfO_2		3.8	9.6×10^{12}	
	16.4 nm $SrTiO_3$	CVD MoS_2	1	4.3×10^{12}	8
Top gate	4.5 nm SiO_2	Si CMOS	4.5	$\sim10^{9}$	16
	2 nm AlO_x/30 nm HfO_2	CVD MoS_2	9.3	1×10^{13}	17
	10 nm HfO_2	exfoliated MoS_2	3.6	5.1×10^{12}	18
	5.3 nm Al_2O_3/TiOPc	exfoliated WSe_2	3	1.1×10^{13}	19
	16 nm HfO_2/BN	exfoliated MoS_2	9.6	7×10^{11}	20
	6.1 nm Al_2O_3	exfoliated MoS_2	3.5	6.6×10^{12}	21
	6.5 nm Ta_2O_5	exfoliated MoS_2	1.64	1.2×10^{12}	22

	7.5nm Al_2O_3/OTS	exfoliated MoS_2	6.1	1.14×10^{12}	23
	16 nm Al_2O_3	exfoliated MoS_2	7.5	3.44×10^{12}	24
	15 nm HfO_2	exfoliated HfS_2	3.9	6×10^{11}	25
	6 nm HfO_2/PTCDA	exfoliated MoS_2	2.1	8×10^{11}	7
	20 nm Y_2O_3	exfoliated MoS_2	4.2	1.36×10^{12}	26
	5.3 nm Er_2O_3	exfoliated MoS_2	1.1	6×10^{11}	1
Top gate	2 nm c-Al_2O_3(1/f)	exfoliated MoS_2	1.1	8.4×10^{9}	This work

Supplementary Table 4. Comparison of SS versus I_{on}/I_{off} of the MoS_2 FET with various dielectrics.

Gates	Channels	Dielectrics	L_{tg} (μm)	ION/IOFF	SS (mV/dec)	EOT (nm)	Refs.
Top gate	exfoliated MoS_2	16 nm Al_2O_3	3	10^8	140		35
	exfoliated MoS_2	20 nm HfO_2	1	10^7	88		33
	exfoliated MoS_2	20 nm Si_3N_4		10^7	95		31
	exfoliated MoS_2	20 nm SiO_2		10^6	200	20	31
	exfoliated MoS_2	13 nm Al_2O_3	0.116	10^7	158		37
	exfoliated MoS_2	9 nm HfO_2/ 4 nm Y_2O_3	0.4	10^8	74	4.4	2
	CVD MoS_2	25 nm Al_2O_3	0.3	10^7	258		36
	exfoliated MoS_2	6.6 nm Al_2O_3	1	10^8	101		34
	exfoliated MoS_2	6 nm HfO_2	0.5	10^7	75		18
	exfoliated MoS_2	16 nm HfO_2/BN	2	10^8	78	9.6	20
	CVD MoS_2	5 nm Al_2O_3/1 nm AlN	1	3×10^6	120		38
	exfoliated MoS_2	6.1 nm Al_2O_3	2	10^9	85	3.5	21
	exfoliated MoS_2	7.5 nm Al_2O_3-OTS	3	10^6	79	4.5	23
	exfoliated MoS_2	3-4 nm HfO_2	0.6	10^6	130		32
	exfoliated MoS_2	4 nm HfO_2		10^6	69		11
	CVD MoS_2	55 nm HfO_2	10	4×10^8	116		28

	exfoliated MoS_2	3 nm HfO_2/PTCDA	4	10^7	60	1.3	7
	exfoliated MoS_2	10 nm Sb_2O_3	1.5	10^8	68	3.3	9
	CVD MoS_2	6-8 nm Al_2O_3	1	10^7	114		29
	exfoliated MoS_2	4 nm HfO_2	2	10^8	63.1		4
	exfoliated MoS_2	20 nm Y_2O_3	1	10^6	60	4.2	26
	CVD MoS_2	10 nm Al_2O_3	5	10^7	68		6
	exfoliated MoS_2	5.3 nm Er_2O_3	2.4	10^6	90	1.1	1
Back gate	exfoliated MoS_2	50 nm Al_2O_3	7	10^6	70		27
	exfoliated MoS_2	30 nm HfO_2	1	10^6	100	6.2	10
	exfoliated MoS_2	5.8 nm ZrO_2	0.001	10^6	65		30
	exfoliated MoS_2	15 nm Al_2O_3		10^6	117	8	11
	CVD MoS_2	8 nm HfO_2/ 1.2 nm TiO_2	1.5	10^8	64	2.4	5
	exfoliated MoS_2	10 nm HfO_2	16	10^6	77.6	4.25	14
	CVD MoS_2	2 nm CaF_2	0.4	10^7	90	0.9	13
	CVD MoS_2	16.4 nm $SrTiO_3$	3.5	10^7	71.5	1	8
	CVD MoS_2	6 nm HfO_2	0.145	10^9	77		39
		12 nm Al_2O_3	6	10^7	108		
Top gate	exfoliated MoS_2	2 nm c-Al_2O_3	2	2.4×10^8	61	1.4	This work
Top gate	exfoliated MoS_2	2 nm c-Al_2O_3	0.25	10^9	76		This work

Supplementary Table 5. Static dielectric constant (*K*), band gap and $\Delta\Phi_{CB}$ of various candidate gate dielectrics on MoS_2.

Materials	K	Band gap(eV)	$\Delta\Phi_{CB}$(eV)	Refs.
MoS_2		2.1		42
c-Al_2O_3 (sapphire)	9	8.8	2.7	43
$SrTiO_3$	40	3.3	0.5	9
SiO_2	3.9	9	3.3	44
a-Al_2O_3	9	6.5	2.5	
HfO_2	23	5.5	2.5	
Mica	8.1	7.85	0.9	
TiO_2	60	3.8	0.4	
Ta_2O_5	15.5	4.4	0.8	
ZrO_x	15	5.8	1.7	
Bi_2SeO_5	21	3.9	2	
CaF_2	8.43	12.1	2.5	

References

1 Muller, D. A. et al. The electronic structure at the atomic scale of ultrathin gate oxides. Nature 399, 758–761 (1999).

2 Uchiyama, H., Maruyama, K., Chen, E., Nishimura, T. & Nagashio, K. A monolayer MoS_2 FET with an EOT of 1.1 nm achieved by the direct formation of a high-k Er_2O_3 insulator through thermal evaporation. Small 19, 2207394 (2023).

3 Zou, X. M. *et al.* Interface engineering for high-performance top-gated MoS_2 field-effect transistors. *Adv. Mater.* 26, 6255-6261 (2014).

4 Lee, B. H., Jeon, Y., Zawadzki, K., Qi, W. J. & Lee, J. Effects of interfacial layer growth on the electrical characteristics of thin titanium oxide films on silicon. *Appl. Phys. Lett.* 74, 3143-3145 (1999).

5 Luo, P. F. *et al.* Molybdenum disulfide transistors with enlarged van der Waals gaps at their dielectric interface via oxygen accumulation. *Nat. Electron.* 5, 849-858 (2022).

6 Zhu, Y. B. *et al.* Monolayer molybdenum disulfide transistors with single-atom-thick gates. *Nano Lett.* 18, 3807-3813 (2018).

7 Lu, Z. Y. *et al.* Wafer-scale high-k dielectrics for two-dimensional circuits via van der Waals integration. *Nat. Commun.* 14, 2340 (2023).

8 Li, W. S. *et al.* Uniform and ultrathin high-k gate dielectrics for two-dimensional electronic devices. *Nat. Electron.* 2, 563-571 (2019).

9 Huang, J. K. *et al.* High-k perovskite membranes as insulators for two-dimensional transistors. *Nature* 605, 262-267 (2022).

10 Liu, K. L. *et al.* A wafer-scale van der Waals dielectric made from an inorganic molecular crystal film. *Nat. Electron.* 4, 906-913 (2021).

11 Wen, C. *et al.* Dielectric Properties of Ultrathin CaF_2 Ionic Crystals. *Adv. Mater.* 32, 2002525 (2020).

12 Ganapathi, K. L., Bhattacharjee, S., Mohan, S. & Bhat, N. High-performance HfO_2 back gated multilayer MoS_2 transistors. *IEEE Electron Device Letters* 37, 797-800 (2016).

13 Bolshakov, P. *et al.* Improvement in top-gate MoS_2 transistor performance due to high quality backside Al_2O_3 layer. *Appl. Phys. Lett.* 111, 032110 (2017).

14 Vu, Q. A. *et al.* Near-zero hysteresis and near-ideal subthreshold swing in h-BN encapsulated single-layer MoS_2 field-effect transistors. *2D Mater.* 5, 031001 (2018).

15 Illarionov, Y. Y. *et al.* Ultrathin calcium fluoride insulators for two-dimensional field-effect transistors. *Nat. Electron.* 2, 230-235 (2019).

16 Pan, Y. *et al.* Near-ideal subthreshold swing MoS_2 back-gate transistors with an optimized ultrathin HfO_2 dielectric layer. *Nanotechnology* 30, 095202 (2019).

17 Smets, Q. *et al.* Ultra-scaled MOCVD MoS_2 MOSFETs with 42 nm contact pitch and 250 μA/μm drain current. *In 2019 IEEE International Electron Devices Meeting (IEDM)* 23.2.1-23.2.4 (IEEE, 2019).

18 Boutchacha, T., Ghibaudo, G., Guégan, G. & Skotnicki, T. Low frequency noise characterization of 0.18 μm Si CMOS transistors. *Microelectron. Reliab.* 37, 1599–1602 (1997).

19 Zhu, W. J. *et al.* Electronic transport and device prospects of monolayer molybdenum disulphide grown by chemical vapour deposition. *Nat. Commun.* 5, 3087 (2014).

20 Wang, J. L. *et al.* Integration of high-k oxide on MoS_2 by using ozone

pretreatment for high-performance MoS_2 top-gated transistor with thickness-dependent carrier scattering investigation. *Small* 11, 5932-5938 (2015).
21 Park, J. H. *et al.* Atomic layer deposition of Al_2O_3 on WSe_2 functionalized by titanyl phthalocyanine. *ACS Nano* 10, 6888-6896 (2016).
22 Zou, X. M. *et al.* Dielectric engineering of a boron nitride/hafnium oxide heterostructure for high-performance 2D field effect transistors. *Adv. Mater.* 28, 2062-2069 (2016).
23 Wang, X. *et al.* Improved integration of ultra-thin high-k dielectrics in few-layer MoS_2 FET by remote forming gas plasma pretreatment. *Appl. Phys. Lett.* 110, 053110 (2017).
24 Chamlagain, B. *et al.* Thermally oxidized 2D TaS_2 as a high-kappa gate dielectric for MoS_2 field-effect transistors. *2D Mater.* 4, 031002 (2017).
25 Cheng, L. X. *et al.* Sub-10 nm tunable hybrid dielectric e ngineering on MoS_2 for two-dimensional material-based devices. *ACS Nano* 11, 10243-10252 (2017).
26 Thakar, K., Varghese, A., Dhara, S., Ghosh, S. & Lodha, S. Thin EOT MoS_2 FET for efficient photodetection and gas sensing. *In 2018 4th IEEE International Conference on Emerging Electronics (ICEE)* 1-4 (IEEE, 2018).
27 Lai, S. *et al.* HfO_2/HfS_2 hybrid heterostructure fabricated via controllable chemical conversion of two-dimensional HfS_2. *Nanoscale* 10, 18758-18766 (2018).
28 Wang, L. Y. *et al.* A general one-step plug-and-probe approach to top-gated transistors for rapidly probing delicate electronic materials. *Nat. Nanotechnol.* 17, 1206-1213 (2022).
29 Kim, S. *et al.* High-mobility and low-power thin-film transistors based on multilayer MoS_2 crystals. *Nat. Commun.* 3, 1011 (2012).
30 Dai, Z. Y., Wang, Z. W., He, X., Zhang, X. X. & Alshareef, H. N. Large-area chemical vapor deposited MoS_2 with transparent conducting oxide contacts toward fully transparent 2D electronics. *Adv. Funct. Mater.* 27, 1703119 (2017).
31 Li, W. *et al.* High-performance CVD MoS_2 transistors with self-aligned top-gate and Bi contact. *In 2021 IEEE International Electron Devices Meeting (IEDM)* 37.3.1-37.3.4 (IEEE, 2021).
32 Desai, S. B. *et al.* MoS_2 transistors with 1-nanometer gate lengths. *Science* 354, 99-102 (2016).
33 Kappera, R. *et al.* Phase-engineered low-resistance contacts for ultrathin MoS_2 transistors. *Nat. Mater.* 13, 1128-1134 (2014).
34 Price, K. M., Schauble, K. E., McGuire, F. A., Farmer, D. B. & Franklin, A. D. Uniform growth of sub-5-nanometer high-k dielectrics on MoS_2 using plasma-enhanced atomic layer deposition. *ACS. Appl. Mater. Interfaces.* 9, 23072-23080 (2017).
35 Wang, H. *et al.* Integrated circuits based on bilayer MoS_2 transistors. *Nano Lett.* 12, 4674-4680 (2012).
36 Yang, W. *et al.* The integration of sub-10 nm gate oxide on MoS_2 with ultra low leakage and enhanced mobility. *Sci. Rep.* 5, 11921 (2015).
37 Liu, H. & Ye, P. D. D. MoS_2 dual-gate MOSFET with atomic-layer-deposited Al_2O_3 as top-gate dielectric. *IEEE Electron Device Letters* 33, 546-548 (2012).
38 Sanne, A. *et al.* Top-gated chemical vapor deposited MoS_2 field-effect transistors on Si_3N_4 substrates. *Appl. Phys. Lett.* 106, 062101 (2015).
39 Cheng, R. *et al.* Few-layer molybdenum disulfide transistors and circuits for

high-speed flexible electronics. *Nat. Commun.* 5, 5143 (2014).
40 Qian, Q. K. *et al.* Improved gate dielectric deposition and enhanced electrical stability for single-layer MoS_2 MOSFET with an AlN interfacial layer. *Sci. Rep.* 6, 27676 (2016).
41 Yang, X. D. *et al.* Highly reproducible van der Waals integration of two-dimensional electronics on the wafer scale. *Nat. Nanotechnol.* 18, 471-478 (2023).
42 Illarionov, Y. Y. et al. Insulators for 2D nanoelectronics: the gap to bridge. *Nat. Commun*. 11, 3385 (2020).
43 Robertson, J. & Wallace, R. M. High-K materials and metal gates for CMOS applications. *Mater. Sci. Eng. R* 88, 1–41 (2015).
44 Knobloch, T. et al. The performance limits of hexagonal boron nitride as an insulator for scaled CMOS devices based on two-dimensional materials. *Nat. Electron*. 4, 98–108 (2021).
